# PEDIATRICIANS AFTER RESIDENCY: A SURVEY OF PERSONAL/PROFESSIONAL DATA AND ISSUES

**DOI:** 10.1590/1984-0462/2021/39/2019190

**Published:** 2020-08-03

**Authors:** Clovis Artur Silva, Vitor Cavalcanti Trindade, Roberta Capretz D’Oliveira Abel, Marcelo Oliveira Silva, João Fernando Vecchi Santos, Vera Hermina Kalika Koch, Ana Paula Scoleze Ferrer, Alexandra Brentani, Vicente Odone-Filho, Uenis Tannuri, Werther Brunow Carvalho, Magda Carneiro-Sampaio, Sandra Josefina Ferraz Ellero Grisi

**Affiliations:** a Universidade de São Paulo, São Paulo, SP, Brazil.

**Keywords:** Pediatrician, Internship and residency, Sedentary behavior, Workload, Medical education, Pediatras, Internato e residência, Comportamento sedentário, Carga de trabalho, Educação médica

## Abstract

**Objective:**

To assess personal, professional, medical, and scientific educational characteristics and issues reported by pediatricians.

**Methods:**

Cross-sectional study based on an online survey including 614 pediatricians who graduated in the last 15 years at a University Pediatric Department in Brazil.

**Results:**

The response rate was 331/614(54%). The majority were females (82%), the median age was 33 years (27-40) and median years of pediatric practice was 5 (1-13). High workload (>60 hours/week) occurred in 25% and 47% earned ≥15 minimum wages/month. The most work-related issues reported were long working hours, poor social life and a sedentary lifestyle (>50%). Pediatricians were further divided into two groups, according to years of pediatric clinical practice: group 1 (≤5 years) and group 2 (>5 years). The median of overall satisfaction with pediatric residency [8(0-10) vs. 9 (4-10); p=0.002] was significantly reduced in group 1. The frequencies of workload >60 hours, work on pediatric ward and pediatric intensive care were significantly higher in the first group (p<0.05). Regarding main issues related to clinical practice in the last year, long working hours (73 vs. 53%; p<0.001), poor social life (75 vs. 62%; p=0.018) and harassment (23 vs. 4%; p=0.003) were significantly higher in the first group.

**Conclusions:**

Very early career pediatricians (≤5 years) reported higher workload, lower income, work-related issues and different location of pediatric practice compared to early career pediatricians (>5 years). The overall satisfaction with pediatric residency was good, however, reduced in very early career pediatricians.

## RESUMO

**Objetivo:**

Avaliar características e problemas pessoais, profissionais, médicos e de educação científica reportados por pediatras.

**Métodos:**

Estudo transversal com base em uma pesquisa online, incluindo 614 pediatras formados nos últimos 15 anos no Departamento de Pediatria de uma universidade brasileira.

**Resultados:**

A taxa de resposta foi de 331/614 (54%). A maioria dos participantes era do sexo feminino (82%), a mediana de idade foi de 33 anos (27-40 anos) e a mediana de tempo de prática pediátrica foi de 5 anos (1-13). Jornada de trabalho elevada (>60 horas/semana) foi relatada por 25% dos entrevistados e 47% recebiam ≥15 salários mínimos/mês. Os problemas relacionados ao trabalho mais frequentes foram jornadas longas de trabalho, vida social insatisfatória e sedentarismo (>50%). Os pediatras foram divididos em dois grupos de acordo com os anos de prática clínica pediátrica: grupo 1 (≤5 anos) e grupo 2 (>5 anos). A mediana de satisfação geral com a residência pediátrica [8 (0-10) versus 9 (4-10); p=0,002] foi significativamente menor no grupo 1. As frequências de jornada de trabalho >60 horas, trabalho na enfermaria pediátrica e na terapia intensiva pediátrica foram significativamente maiores no primeiro grupo (p<0,05). Quanto aos principais problemas relacionados à prática clínica no ano anterior, jornadas longas de trabalho (73 versus 53%; p<0,001), vida social insatisfatória (75 versus 62%; p=0,018) e assédio (23 versus 4%; p=0,003) foram significativamente mais elevados no grupo 1.

**Conclusões:**

Pediatras em início de carreira (≤5 anos) relataram maior jornada de trabalho, menor renda, problemas relacionados ao trabalho e diferentes locais de trabalho em comparação com pediatras mais experientes (>5 anos). A satisfação geral com a residência pediátrica foi boa, porém menor em pediatras do primeiro grupo.

## INTRODUCTION

General and pediatric residency programs have been modified worldwide with new curriculums, including a modern approach to teaching, patient care with ethical and humanistic attitudes, and research training.^1^ ^,^ ^2^ ^,^ ^3^ ^,^ ^4^ ^,^ ^5^ ^,^ ^6^ Increasing technological innovation with new medical procedures, radiology and laboratory exams, translational medicine with drugs development and specific therapeutic targets for each disease are a reality in daily pediatric practice.^2^ ^,^ ^5^ ^,^ ^7^ ^,^ ^8^ ^,^ ^9^ ^,^ ^10^ ^,^ ^11^ ^,^ ^12^ ^,^ ^13^

After the conclusion of pediatric residency, pediatricians and pediatric specialists routinely deal with different patient care profiles, including life-threatening, chronic diseases, patients suffering and death.^8^ ^,^ ^9^ In addition, severely compromised patients, harassment, discrimination, low income and disruption in family life may induce high levels of physical/psychological stress and/or professional issues in pediatricians^8^ ^,^ ^14^ ^,^ ^15^ ^,^ ^16^ ^,^ ^17^ and may reduce their overall satisfaction,^18^ particularly in very early career physicians.^19^

To the best of our knowledge, simultaneous analysis of personal, professional, medical, and scientific educational characteristics and issues reported by pediatricians have not been carried out in Latin America.

Therefore, this study aimed to assess pediatricians’ reports regarding demographic data, location of clinical practice, pediatric specialties, overall satisfaction rates with residency/clinical practice, income, medical and scientific education, patient care profiles, laboratory exams/medication use and main physical, psychiatric/psychological and professional issues related to clinical practice. In addition, comparisons of personal, professional, educational characteristics and issues according to years of pediatric clinical practice were evaluated.

## METHOD

A cross-sectional study involving 614 pediatricians was carried out based on an online survey in terms of personal, professional, medical, and scientific educational characteristics and issues. All physicians had successfully concluded the General Pediatric Residency Program and/or Pediatric Specialties Residency Program in a Pediatric Department in Brazil, which are teaching-oriented training programs. The General Pediatric Residency Program includes primary and secondary care activities during the first year of training, focusing on care and attention to healthy children and adolescents. In the second and third years, there is a predominance of secondary and tertiary care activities at outpatient and inpatients clinics, including multiple pediatric subspecialties.

The survey was carried out using REDCap tool, which is a secure web application for building, managing, and accessing electronic questionnaires and databases. This survey was sent to all of the pediatricians between November 2018 and January 2019. They had concluded their General Pediatric Residency Program or Pediatric Specialties Residency Program between 2006 and 2018. At least 15 emails were sent to improve the response survey rate. The Ethics Committee of our university hospital approved this study (CAAE: 93564518.5.0000.0068) and an informed consent was obtained from all participants.

Anonymous self-report questionnaire invitations were distributed by email. The online survey comprised 21 questions focused on physician-reported personal, educational, and professional issues. These questions were multiple-choice, dichotomous (yes and no) or horizontal visual analog scale, recollecting events during their practice. Estimated time for completion was nearly 15 minutes

The online survey included 21 items related to the following issues:

1. Demographic data of pediatricians (current age, gender, marital status, number of children, city, state, country and years of pediatric clinical practice after the conclusion of general pediatric residency).
2. Location of pediatricians practice in the last year (public service, private service, pediatric primary care, pediatric ward, pediatric emergency room, pediatric intensive care, neonate care, pediatrician private practice, public/private university professor, pharmaceutical industry, medical procedure, radiology service, laboratory tests, non-governmental organization and administrative service).
3. Pediatric specialties after General Pediatric Residency Program (Adolescent Medicine, Cardiology, Developmental and Behavioral, Emergency, Endocrinology, Gastroenterology, Genetics, Hematology, Pediatric Intensive Care, Immunology and Allergy, Infectious Disease, Neonatology, Nephrology and Renal Transplantation, Neurology, Nutrology, Oncology, Palliative and Pain Care, Pneumology, Rheumatology and no specialty).
4. Overall satisfaction rate during General Pediatric Residency Program at the Pediatric Department of Universidade de São Paulo, measured in a horizontal visual analog scale (0 = no satisfaction at all and 10=excellent overall satisfaction).
5. Overall satisfaction rate with pediatric clinical practice in the last year, measured in a horizontal visual analog scale (0=no satisfaction at all and 10=excellent overall satisfaction).
6. Workload in hours/week in the last year (≤20 hours, 20-40 hours, 40-60 hours and >60 hours).
7. Number of pediatric patients/week in the last year (≤50 patients, 50-100 patients, 100-200 patients, and >200 patients).
8. Health care insurance availability for pediatric patients in the last year (public health insurance, private insurance and/or other).
9. Pediatrician income in the last year (<15 minimum wages/month and ≥15 minimum wages/month).
10. Work exclusively as a pediatrician in the last year (yes/no).
11. Scientific initiation fellowship during medical school (yes/no).
12. Number of meeting/congress/course participation in the last year.
13. Published papers in medical literature (yes/no).
14. Enrolled in Master’s and/or PhD (doctor of philosophy) program (yes/no).
15. Main patient care profiles in the last year (emergency room, ward, pediatric intensive care, primary care, newborn, growth and development monitoring, chronic disease management, psychiatric/psychological management, contraception and gynecological counseling, licit and illicit drug use, sexually transmitted infections and pregnancy counseling, and physical/psychological violence assessment).
16. Use of laboratory tests during clinical practice in the last year (clinical laboratory, radiography, electrocardiogram, electroencephalogram, endoscopic examinations, echocardiogram, ultrasonography, computed tomography, magnetic resonance imaging, bone densitometry, eye examination, biopsy, whole genomic sequencing, and audiometry).
17. Medication use during clinical practice in the last year (antibiotic, non-hormonal anti-inflammatory, oral or intravenous glucocorticoid, inhaled glucocorticoid, vasoactive drug, surfactant, antidepressant, anticonvulsant, painkiller, antihistamine, immunosuppressive/chemotherapy, immunobiological, contraceptive, vaccines, and homeopathy).
18. Contraception prescription for adolescent patients in the last year (condom, oral contraceptive, depot medroxyprogesterone acetate, implant progesterone contraceptive, emergency contraception, intrauterine device and/or none of them).
19. General supportive care used during clinical practice in the last year (electronic medical chart, day hospital, anthropometric data evaluation, health-related quality of life questionnaires, pain assessment evaluation, dental care, physiotherapy, dietary orientation, speech and language, psychological support, rehabilitation care therapy, sex education, physical activity orientation, patients/family education about the illness and treatment, multidisciplinary team approach, phone calls to improve adherence and schedule appointments, participation in clinical trials, adverse events monitoring, sunscreen protection, palliative care support and transition-focused program to adult care).
20. Vaccination card evaluation in all appointments in the last year (yes/no).
21. Main physical, psychiatric, psychological, and professional issues related to clinical practice in the last year (long working hours, poor social life, physical inactivity, overall decrease of health-related quality of life, anxiety, depression, burnout syndrome, disruption in family life, harassment, obsessive-compulsive symptoms, low income, legal issues, workplace violence, stress induced by boss/professor and stress induced by health insurance).

Pediatricians were further subdivided into two groups according to the median years of pediatric clinical practice: group 1 (≤5 years) and group 2 (>5 years).

The *Statistical Package for the Social Sciences* version 13.0 was used. The results for the continuous variables were presented by median (minimum-maximum values) or mean±standard deviation (SD), and compared by Mann-Whitney test and Student’s t-test, respectively. The results for categorical variables were presented as frequency (percentage) and compared by Fisher’s exact test or Pearson chi-square test, as appropriated. Pearson or Spearman rank correlation coefficients were used for correlations between overall satisfaction with pediatric residency/pediatrician clinical practice and current age and years of medical practice after residency conclusion. P values less than 0.05 were considered significant.

## RESULTS

The response rate for this web-based questionnaire was 377/614 (61%) pediatricians. Incomplete data were observed in 44/614 (7%) respondents and only 2/614 (0.3%) declined to participate in the present study. Therefore, 331/614 (54%) respondents completed the questionnaire and were evaluated.

Table 1 shows demographic data, the location of practice and type of pediatric specialties reported by pediatricians after residency conclusion. Most pediatricians were female (82%), with median of current age 33 years (27-40) and median of 5 years of pediatric clinical practice after residency program (1-13). The main local of practice reported by pediatricians in the last year were public (79%) and private services (81%), whereas medical procedure, administrative service, public university professor, radiology service, laboratory, pharmaceutical industry, and non-governmental organization were infrequently described (<4%). The three most frequent pediatric specialties reported by respondents were Neonatology (25%), Pediatric Intensive Care (14%) and Pediatric Cardiology (7%) (Table 1).

**Table 1:** Demographic data, location of practice and type of pediatric specialties reported by pediatricians after residency conclusion.

	Pediatricians (n=331)
Demographic data	
Current age, years	33 (27-40)
Female	272 (82)
Years of medical practice after residency program	5 (1-13)
Current married/partnered	217 (65)
Number of children	0 (0-4)
Location of practice in the last year	
Public service	262 (79)
Private service	269 (81)
Pediatric primary care	16 (5)
Pediatric ward	96 (29)
Pediatric emergency room	142 (43)
Pediatric intensive care	107 (32)
Neonate care	63 (19)
Pediatrician private practice	219 (66)
Public university professor	11 (3)
Private university professor	22 (7)
Pharmaceutical industry	2 (1)
Medical procedure	13 (4)
Radiology service	8 (2)
Laboratory	3 (1)
Non-governmental organization	4 (1)
Administrative service	12 (4)
Type of pediatric specialties	
Adolescent Medicine	2 (1)
Cardiology	22 (7)
Developmental and behavioral	3 (1)
Emergency	13 (4)
Endocrinology	15 (4)
Gastroenterology	11 (3)
Genetics	1 (0.3)
Hematology	7 (2)
Pediatric intensive Care	45 (14)
Immunology and Allergy	24 (7)
Infectious disease	8 (2)
Neonatology	71 (25)
Nephrology and Renal Transplantation	17 (5)
Neurology	10 (3)
Nutrology	1 (0.3)
Oncology	21 (6)
Palliative and Pain Care	1 (0.3)
Pneumology	14 (4)
Rheumatology	8 (2)
No specialty	37 (11)

Results are presented in n (%) and median (range) or mean±standard deviation.

Most pediatricians (86%) followed-up patients aged between 28 days to 10 years. High workload (>60 hours/week) occurred in one-quarter of pediatricians, and 15% examined more than 100 pediatric patients/week. Almost 50% of pediatricians earned ≥15 minimum wages/month. At least one meeting/congress/course participation was reported in almost 90% of physicians, and approximately 30% reported published research papers in medical literature. Scientific initiation fellowship during medical school was described by 64% of respondents, and 16% reported enrollment in Master and/or PhD program (Table 2).

**Table 2:** Patient age groups, workload/week, number of patients/week, pediatrician’s income, work exclusively as a pediatrician, scientific initiation fellowship during medical school, number of meeting/congress/course participation, published papers and enrolled in Master’s and PhD programs reported by pediatricians in the last year, after residency conclusion.

	Pediatricians (n=331)
Patient age groups	
Newborn	245 (74)
28 days to <10 years	285 (86)
10-20 years	216 (65)
>20 years	44 (13)
Workload hours/week	
<20 hours	30 (9)
20-40 hours	78 (24)
40-60 hours	139 (42)
>60 hours	84 (25)
Number of pediatric patients/week	
<50 patients	166 (50)
50-100 patients	115 (35)
100-200 patients	29 (9)
>200 patients	21 (6)
Pediatrician’s income	
≥15 minimum wages/month	157 (47)
Work exclusively as a pediatrician	262 (79)
Scientific initiation fellowship during medical school	211 (64)
Number of meeting/congress/course participation	
1-2	178 (54)
3-5	95 (29)
>6	20 (6)
None	38 (11)
Published research papers in medical literature	88 (27)
Enrolled in Master’s and/or PhD program	54 (16)

Results are presented in n (%); PhD: doctor of philosophy.

The main patient care profiles described by pediatricians were emergency room, pediatric intensive care, ward, and growth and development monitoring (>40%). The most important physical, psychiatric/psychological and professional issues (>50%) were long working hours, poor social life, physical inactivity and disruption in family life (Table 3).

**Table 3:** Main patient care profiles, laboratory exams, medication use, contraception prescription, general supportive care, vaccination card evaluation in all appointments and main physical, psychiatric/psychological and professional issues reported by pediatricians in the last years after residency conclusion

	Pediatricians (n=331)
Patient care profiles	
Emergency room	169 (51)
Pediatric ward	135 (41)
Pediatric intensive care	151 (46)
Primary care	41 (12)
Newborn	109 (33)
Growth and development monitoring	138 (42)
Chronic disease management	115 (35)
Psychiatric/psychological management	15 (4)
Contraception and gynecological counseling	2 (1)
Licit and illicit drug use	3 (1)
STI and pregnancy counseling	3 (1)
Physical/psychological violence assessment	7 (2)
Laboratory exams	
Clinical laboratory	313 (95)
Radiography	289 (87)
Ultrasonography	243 (73)
Medication use	
Antibiotic	315 (92)
Oral or intravenous glucocorticoid	296 (89)
Painkiller	264 (80)
Antihistamine	224 (68)
Contraception prescription for adolescent	
Condom	56 (17)
Oral progesterone	42 (13)
Emergency contraception	4 (10)
General supportive care	
Electronic medical chart	271 (82)
Anthropometric data evaluation	238 (72)
Physiotherapy	229 (69)
Vaccination card evaluation in all appointments	198 (60)
Physical, psychiatric/psychological and professional issues	
Long working hours	209 (63)
Poor social life	187 (56)
Physical inactivity	203 (61)
Overall decrease of HRQL	153 (46)
Anxiety	147 (44)
Depression	51 (14)
Burnout syndrome	105 (32)
Disruption in family life	195 (59)
Harassment	29 (9)
Obsessive compulsive symptoms	14 (4)
Low income	105 (32)
Legal problem	10 (3)
Workplace violence	9 (3)
Stress induced by boss/professor	99 (30)
Stress induced by health insurance	36 (11)

Results are presented in n (%); STI: sexually transmitted infections; HRQL: health-related quality of life.

Groups were further divided into two: Group 1 [≤5 years of clinical practice after conclusion of general pediatric residency, n=172/302 (67%)] and group 2 [>5 years, n=159/312 (56%)]. The median of current age [30 (27‒37) vs. 35 (31‒40) years; p<0.001] and number of children [0 (0‒3) vs. 1 (0‒4); p<0.001] were significantly lower in group 1 compared to group 2 (Table 4). The median of overall satisfaction with pediatric residency [8 (0‒10) vs. 9 (4‒10); p=0.002] was significantly higher in group 2, however with a similar median of overall satisfaction with pediatric clinical practice [8 (1‒10) vs. 8 (4‒10); p=0.845] (Table 5).

**Table 4:** Demographic data and type of pediatric specialties according to years of pediatric clinical practice in two groups: group 1 (≤5 year) and group 2 (>5 year).

	group 1 (n=172)	group 2 (n=159)	p-value
Demographic data			
Current age, years	30 (27-37)	35 (31-40)	<0.001
Female	136 (79)	136 (85)	0.151
Pediatrician practice duration, years	3 (1-5)	8 (6-13)	<0.001
Currently married/partnered	89 (52)	128 (80)	<0.001
Number of children	0 (0-3)	1 (0-4)	<0.001
Type of pediatric specialties			
Adolescence Medicine	1 (1)	1 (1)	1.000
Cardiology	9 (5)	15 (9)	0.202
Developmental and behavioral	3 (2)	0 (0)	0.249
Emergency	6 (3)	7 (4)	0.780
Endocrinology	6 (3)	9 (6)	0.431
Gastroenterology	8 (5)	3 (2)	0.223
Genetics	0 (0)	1 (1)	0.480
Hematology	4 (2)	3 (2)	1.000
Pediatric intensive care	19 (11)	26 (16)	0.199
Immunology and allergy	9 (5)	15 (9)	0.202
Infectious disease	5 (3)	3 (2)	0.725
Nephrology and renal transplantation	8 (5)	9 (6)	0.805
Neonatology	30 (17)	41 (26)	0.081
Neurology	5 (3)	5 (3)	1.000
Nutrology	0 (0)	1 (1)	0.480
Oncology	8 (5)	13 (8)	0.259
Palliative and pain care	1 (1)	0 (0)	1.000
Pneumology	11 (6)	3 (2)	0.055
Rheumatology	4 (2)	4 (2)	1.000
No specialty	32 (19)	5 (3)	<0.001

Results are presented in n (%) and median (range).

**Table 5:** Location of practice, workload, number of patients/week, overall satisfaction with pediatric residency/clinical practice, meeting/congress/course participation, published papers and main issues related to profession according to years of pediatric clinical practice in two groups: group 1 (≤5 year) and group 2 (>5 year).

	group 1 (n=172)	group 2 (n=159)	p-value
Location of pediatric practice in the last year			
Public service	139 (81)	123 (77)	0.499
Private service	143 (83)	126 (79)	0.399
Pediatric primary care	8 (5)	8 (5)	1.000
Pediatric ward	64 (37)	32 (20)	0.001
Pediatric emergency room	99 (58)	43 (27)	<0.001
Pediatric intensive care	54 (31)	53 (33)	0.725
Neonate care	31 (18)	32 (20)	0.675
Pediatrician private practice	75 (44)	90 (57)	0.021
Workload hours/week			
≤20 hours	15 (9)	15 (9)	0.850
20-40 hours	31 (18)	47 (30)	0.014
40-60 hours	72 (42)	67 (42)	1.000
>60 hours	54 (31)	30 (19)	0.011
Pediatric patients/week			
≤50 patients	79 (46)	87 (55)	0.124
50-100 patients	66 (38)	49 (31)	0.166
100-200 patients	13 (8)	16 (10)	0.443
>200 patients	14 (8)	7 (4)	0.182
Pediatrician’s income			
≥15 minimum wages/month	57 (33)	100 (63)	<0.001
Overall satisfaction with pediatric residency, VAS score	8 (0-10)	9 (4-10)	0.002
Overall satisfaction with clinical practice, VAS score	8 (1-10)	8 (4-10)	0.845
Meeting/congress/course participation in last year	153 (89)	140 (88)	0.864
Published papers	49 (28)	39 (24)	0.456
Main issues related to clinical practice in last year			
Long working hours	125 (73)	84 (53)	<0.001
Poor social life	128 (75)	99 (62)	0.018
Anxiety or depression	84 (49)	73 (46)	0.659
Burnout syndrome	60 (35)	45 (28)	0.237
Harassment	23 (13)	6 (4)	0.003
Low salary	49 (28)	56 (35)	0.196

Results are presented in n (%) and median (range); VAS: visual analogy scale (0=no and 10=excellent overall satisfaction).

The frequencies of general pediatricians (19 vs. 3%; p<0.001), workload >60 hours (31 vs. 19%; p=0.011), work on pediatric ward (37 vs. 20%; p=0.001), and pediatric intensive care (58 vs. 27%; p<0.001) were significantly higher in group 1 compared to group 2, whereas being currently married/partnered (52 vs. 80%; p<0.001) and work on private practice were significantly lower in the first group (44 vs. 57%; p=0.021). Regarding main issues related to clinical practice in the last year, long working hours (73 vs. 53%; p<0.001), poor social life (75 vs. 62%; p=0.018) and harassment (23 vs. 4%; p=0.003) were significantly higher in the former group (Tables 4 and 5).

Regarding Spearman’s rank correlations, current age and years of pediatric clinical practice after completing residency were not correlated with overall satisfaction with pediatric residency and overall satisfaction with pediatric clinical practice (p>0.05).

Further analyses were also carried out comparing two groups according to the duration of pediatric residency program: A (3 years of pediatric residency program, n=82) and B (2 years of pediatric residency program, n=249). The frequencies of infirmary (45 vs. 24%; p<0.001) and emergency room (77 vs. 32%; p<0.001) were significantly higher in group A than in B. The median of overall satisfaction with pediatric residency [8 (0-10) vs. 8 (4-10); p=0.015] and the frequency of pediatricians with ≥15 minimum wages/month salary (13 vs. 59%; p<0.001) were significantly higher in group B than A, as well as pediatric specialty (68 vs. 96%; p<0.001).

## DISCUSSION

This was the first web-based survey to carry out a simultaneous analysis of work-life balance and scientific education of pediatricians after the conclusion of residency in a Latin America Residency Program. Pediatricians were young, reported low income and high workload, particularly in emergency room and pediatric ward. The overall satisfaction with pediatric residency was good, however, reduced in very early career pediatricians (≤5 years).

The advantage of the present study was the moderate response rate (54%) of the online self-reported survey without financial incentive. This response rate observed herein contrasted with a low response in previous studies with neonatologists, members of The American Academy of Pediatrics (15%),^20^ with undergraduates in neurology residency (23%)^13^ and with medical students, residents and early career physicians listed in Physician Masterfile (35%).^17^ The confidentiality of the survey was relevant since there was no disclosure of their identity. Another strength of this study was the assessment of self-report and standardized questionnaire, including measuring instruments to assess overall satisfaction.

Pediatricians in the present study were young, married, and with a low number of children. A predominance of female sex was also observed, similar to other studies.^18^ ^,^ ^21^ Indeed, this finding is related to the increase of women in medical schools and residency programs around the world.^22^ ^,^ ^23^

Nearly 80% of pediatricians worked in both public and private services, known as dual practice. This is a particularity in Brazil, where more than half of medical professionals are engaged in dual practice.^24^

Our residents were exposed to all pediatric specialties during the residency training program. However, the most frequent pediatric specialties chosen by our pediatricians were Neonatology, Pediatric Intensive Care and Pediatric Cardiology, as also previously reported.^11^ In fact, these specialties are hospital and procedure-based and may have more job opportunities with an improvement of income.^11^ ^,^ ^25^

Of note, the medical profile described in our institution may not represent the Brazilian pediatrician demographic data. In fact, data from the state of Sao Paulo in 2018 showed that the mean age of pediatricians was 47.4±11.6 years, and the most frequent pediatric specialties were Immunology and Allergy, Oncology and Endocrinology.^26^

Regarding continuing medical educational, the majority of pediatricians attends medical conferences regularly. This result was important to physicians due to great advances in scientific knowledge in clinical practice in newborn, children and adolescent patients, particularly in those with complex chronic diseases.^2^ ^,^ ^3^ ^,^ ^4^ ^,^ ^12^ ^,^ ^27^ One-quarter of pediatricians reported publishing research papers in medical literature. There has been an increase of this practice in our Medical School and Pediatric Department in the last years, reinforcing the relevance of additional gains in writing a manuscript,^28^ such as professional education, financial, social benefits and intellectual pleasure. This is also relevant for those residents that intend to engage in academic medicine.

Importantly, the main location of pediatric practice described by very early career pediatricians was emergency room and pediatric ward, requiring long working hours with poor social life and sedentary lifestyle. The possibility of acute, severely compromised, life-threatening and chronic patients may induce issues and contribute to the decrease in overall satisfaction with pediatric residency reported by our pediatricians with less than 5 years of clinical practice.

In addition, these are issues in medical training programs around the world. The transition from residency training to clinical practice may be challenging for very early career pediatrician, causing work-related stress, fatigue, discomfort and emotional exhaustion, such as burnout, anxiety, depression and susceptibility to harassment.^14^ ^,^ ^16^ ^,^ ^17^

Our pediatricians with >5 years of clinical practice became more specialized, worked at private practices and reported high income. Indeed, self-employed pediatricians may choose their workload based on preferences and financial interests.^29^ ^,^ ^30^

The differences between the location of pediatric practices among the pediatricians showed that most physicians started their early career in the emergency rooms and wards and, later on, in pediatric specialties, particularly working in pediatrician private practice. This reinforces the need for the residency programs to cover all areas of practice in pediatrician training.

Of note, the overall satisfaction with clinical practice was high and similar in very early (≤5 years) and early career pediatricians (>5 years). Our study suggests that pediatricians are pleased with their profession, probably due to the strict relationship between patients and family, and the holistic awareness on mental, physical and emotional health. However, general pediatrics is non-procedure based, contrasting with early-career cardiologists training in Japan, where satisfaction was associated with invasive procedures, such as coronary angiography and percutaneous coronary interventions.^19^

In 2014, a new 3-year pediatric residency program was instituted in our Pediatric Department, aimed at training pediatricians in the 21^st^ century. The topics of Adolescent Medicine, Developmental and Behavioral Care, mental health and pediatric chronic diseases care were expanded on. Approximately one third of our 3-year pediatric residents did not study pediatric specialties after the general residency program. Future multicenter studies with a large population will be necessary to clarify this issue.

The limitations of this study included the possibility of memory bias, since respondents were asked to report questions preferable using a 1-year recall period. In addition, the results of this survey must not be generalized beyond the pediatrician population in our country. Other limitations were the cross-sectional study design, and the fact that the survey did not include instruments to evaluate physiological/psychiatric issues. Therefore, longitudinal and qualitative studies are needed to clarify the trajectory of pediatricians and gain more insight into work-life balance and future goals.

In conclusion, very early career pediatricians (≤5 years) reported higher workload, lower income, and work-related issues compared with late career pediatrician (>5 years), but over time they moved on to private practices and specialized care, consequently improving earnings and living conditions. The overall satisfaction with pediatric residency was good, but reduced in very early career pediatricians, possibly due to not being able to encompass all the knowledge they had acquired in their medical residency.
